# Integration of Pediatric Palliative Care in Oncology: A Scoping Review

**DOI:** 10.1016/j.jpedcp.2026.200205

**Published:** 2026-03-09

**Authors:** Stefanie Stober, Sebastian Hoffmann, Sabine Metzing

**Affiliations:** 1 Department of Nursing Science, Faculty of Health, Witten/Herdecke University, Witten, Germany; 2 Department of Human Medicine, Faculty of Health, Witten/Herdecke University, Witten, Germany

**Keywords:** evaluation, implementation, palliative care, pediatric nursing, pediatric oncology, scoping review

## Abstract

**Objective:**

Pediatric palliative care (PPC) enhances quality of life for children with cancer and their families, yet its systematic integration into oncology practice remains limited, particularly in hospital-based care. This scoping review aimed to map and synthesize international evidence on how PPC is designed, implemented, and evaluated in pediatric oncology inpatient settings, and to identify key barriers and facilitators to its clinical integration.

**Methods:**

We conducted a comprehensive search of MEDLINE, CINAHL, PsycINFO, and Web of Science (2014-2025). Reporting followed PRISMA-ScR standards. Data were charted and narratively synthesized.

**Results:**

Thirty-four studies from 18 countries met inclusion criteria. Most used qualitative methods (n = 12), followed by retrospective chart reviews (n = 7), surveys (n = 4), and reviews or guidelines (n = 11). Evidence revealed delayed initiation of PPC, heterogeneous service models, and limited outcome evaluation. Seven recurrent barriers emerged: absence of standardized models, structural constraints, inadequate staff training, suboptimal communication and family engagement, emotional and cultural barriers, psychosocial strain, and insufficient evaluation frameworks.

**Conclusions:**

These findings highlight a global need to integrate early, family-centered PPC within pediatric oncology inpatient care. Health systems should prioritize staff training, establish adaptable interdisciplinary models, and implement structured evaluation strategies to strengthen the quality and consistency of PPC delivery.

## Highlights

• PPC in pediatric oncology is often ntroduced late and inconsistently.
• Satffing gaps, funding issues, and absent refferal pathways delay PPC.
• International and European PPC standards are unevenly implemented.
• Survey data show marked heterogeneity in PPC models across Europe.
• Early, family-centered PPC models are urgently needed in inpatient care.

## Graphical abstract

**Figure:**
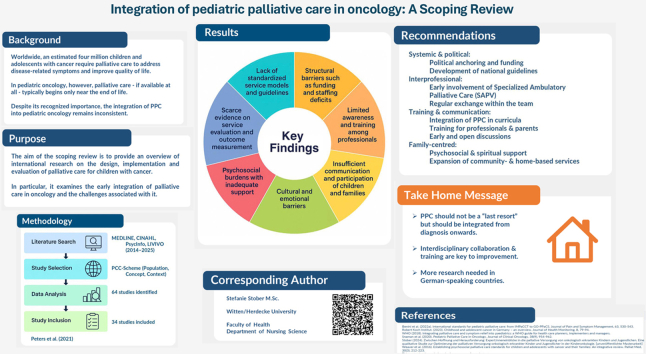

Worldwide, an estimated 4 million children and adolescents with cancer require palliative care to address disease-related symptoms and improve quality of life.^1^ Although survival rates for pediatric cancers are higher than in adults, cancer remains a leading cause of childhood mortality.^2^ According to the World Health Organization, many patients globally still lack access to palliative care, despite evidence that early provision can alleviate suffering, enhance quality of life, and support coping capacities for both patients and families.^3^ In pediatric oncology; however, palliative care—if available at all—typically begins only near the end of life.^1^^,^^4^

Pediatric palliative care (PPC) extends beyond symptom management by integrating psychological, social, and spiritual dimensions into care. While sharing core principles with adult palliative care, PPC requires adaptation to the child's developmental stage and emphasizes the family's central role.^5^^,^^6^ Children are not little adults: they require individualized, developmentally appropriate care, and PPC particularly emphasizes the needs of the entire family unit.^7^

Despite its recognized importance, the integration of PPC into pediatric oncology remains inconsistent. Specific challenges arise from the unique culture of pediatric oncology, where curative treatment often dominates and palliative approaches are introduced late.^6^^,^^8^ Existing reviews have predominantly addressed PPC for children with life-limiting conditions in general^1^^,^^7^, and systematic syntheses focusing specifically on pediatric oncology are scarce. Evidence describing how PPC services are designed, implemented, and evaluated in oncology settings remains limited and fragmented.^9^^,^^10^

## Aim and Research Question

The aim of this scoping review was to systematically map and synthesize international evidence on the design, implementation, and evaluation of PPC in pediatric oncology inpatient settings. Because inpatient care often involves more complex cases and enables multidisciplinary interventions, the focus was placed on this context.

This review was guided by the following overarching research question: What is the current international evidence on the design, implementation, and evaluation of PPC in pediatric oncology inpatient settings? To structure the data extraction and synthesis, the following subquestions were defined.

• Design: What structural models and care concepts of PPC are described in pediatric oncology inpatient settings?
• Implementation: How is PPC implemented in pediatric oncology inpatient settings?
• Evaluation: What approaches and results regarding the evaluation of PPC in pediatric oncology inpatient care are reported?
• Barriers and facilitators: What barriers and facilitators to the implementation of PPC in pediatric oncology inpatient settings are described?

## Methods

### Framework and Reporting

This scoping review followed the methodological framework developed by Arksey and O'Malley^11^ and further consolidated in the Joanna Briggs Institute (JBI) Manual for Evidence Synthesis^12^ Reporting was guided by the PRISMA-ScR checklist.^13^ The review protocol was preregistered on the Open Science Framework (OSF registration link https://osf.io/ks5h3/).

### Eligibility Criteria

Eligibility was defined using the Population, Concept, Context framework recommended by the JBI.^12^

• Population: Children and adolescents (≤18 years) with cancer.
• Concept: Studies addressing the design, implementation, or evaluation of PPC in oncology.
• Context: Inpatient pediatric oncology settings.

Additional inclusion criteria were publication between January 2014 and April 2025 (chosen to capture contemporary developments following the establishment of structured PPC programs^1^), publication in English or German, and availability of full text. Exclusion criteria were studies not specific to pediatric oncology, outpatient-only interventions, or publications without original data (unless providing relevant standards or models).

### Information Sources and Search Strategy

Systematic searches were conducted in MEDLINE (via PubMed), CINAHL, PsycINFO, and Web of Science until April 2025. The search strategy combined controlled vocabulary (eg, MeSH terms) and free-text terms for children, oncology, and palliative care. An example of the electronic search strategy is presented (Table I). Reference lists of all included studies and relevant reviews^6^^,^^14^ were hand searched for additional records.

**Table I:** Search strategy using MEDLINE (PubMed) as an example (own representation)

Search components	No.	Keywords	Number of matches
Pediatric palliative care	#1	((“Palliative Care”[Mesh]) OR “Hospice and Palliative Care Nursing”[Mesh]) OR “Palliative Medicine”[Mesh]	67 792
	#2	“Pediatric Palliative Care”	1653
	#3	“Pediatric Palliative Care Concept”	289
	#4	“Palliative Nursing Care”	80
	#5	“Palliative Nursing”	615
	#6	“Terminal care”	34 780
	#7	“Palliative Therapy”	2297
	#8	“Cancer palliative Therapy”	4
	#9	“Palliative Medicine”	22 640
	#10	“Pediatric and Palliative care”	8581
	#11	“Pediatric nursing”	18 509
	#12	“Pediatric end-of-life care”	22
	#13	“Pediatric Palliative care nursing”	1822
	#14	“Hospice care cancer”	7861
	#15	#1-#14OR ((((((((((((("Pediatric Palliative Care") OR ("Pediatric Palliative Care Concept")) OR ("Palliative Nursing Care")) OR ("Palliative Nursing")) OR ("Terminal care")) OR ("Palliative Therapy")) OR („Cancer palliative Therapy")) OR ("Palliative Medicine")) OR ("Pediatric and Palliative care")) OR ("Pediatric nursing")) OR ("Pediatric end-of-life care")) OR ("Pediatric Palliative care nursing")) OR ("Hospice care cancer")) OR ((("Palliative Care"[Mesh]) OR "Hospice and Palliative Care Nursing"[Mesh]) OR "Palliative Medicine"[Mesh])	130112
Children and adolescents up to 18 years of age with an oncological disease	#16	“Pediatric malign∗ diseas∗”	18
	#17	“Child∗ with cancer”	6151
	#18	“Teenage∗ with cancer”	2512
	#19	“Young people with cancer”	166
	#20	“Adolescents with cancer”	978
	#21	“Life-limited oncological disease”	1
	#22	“Life-limited oncological illness”	0
	#23	Malign∗	777 389
	#24	#15-#22 OR ((((((((Malign∗) OR ("life limited"[All Fields] AND ("oncologic"[All Fields] OR "oncological"[All Fields] OR "oncologically"[All Fields] OR "oncologics"[All Fields]) AND ("illness"[All Fields] OR "illness s"[All Fields] OR "illnesses"[All Fields]))) OR ("Life-limited oncological illness")) OR ("Life-limited oncological disease")) OR ("Adolescents with cancer")) OR ("Young people with cancer")) OR ("Teenage∗ with cancer")) OR ("Child∗ with cancer")) OR ("Pediatric malign∗ diseas∗")	785 660
Pediatric haematology and oncology	#25	“Pediatric oncology ward”	39
	#26	“Pediatric Hematology and Oncology”	12 168
	#27	“Pediatric Oncology unit”	1083
	#28	“Oncology ward in the clinic”	5224
	#29	“Pediatric cancer ward”	899
	#30	“Children's cancer ward”	3
	#31	“Pediatric cancer unit”	5
	#32	“Child∗ Oncology”	2652
	#33	#24-#31 OR ((((((("Child∗ Oncology") OR ("Pediatric cancer unit")) OR ("Childrens cancer ward")) OR ("Pediatric cancer ward")) OR ("Oncology ward in the clinic")) OR ("Pediatric Oncology unit")) OR ("Pediatric Hematology and Oncology")) OR ("Pediatric oncology ward")	21 231
	#34	"Pediatrics"[Mesh]	64 967
	#35	#33 OR #34 ("Pediatrics"[Mesh]) OR (((((((("Child∗ Oncology") OR ("Pediatric cancer unit")) OR ("Childrens cancer ward")) OR ("Pediatric cancer ward")) OR ("Oncology ward in the clinic")) OR ("Pediatric Oncology unit")) OR ("Pediatric Hematology and Oncology")) OR ("Pediatric oncology ward"))	85 923
Total	#36	#15 AND #24 AND #35 ((((((((((Malign∗) OR ("life limited"[All Fields] AND ("oncologic"[All Fields] OR "oncological"[All Fields] OR "oncologically"[All Fields] OR "oncologics"[All Fields]) AND ("illness"[All Fields] OR "illness s"[All Fields] OR "illnesses"[All Fields]))) OR ("Life-limited oncological illness")) OR ("Life-limited oncological disease")) OR ("Adolescents with cancer")) OR ("Young people with cancer")) OR ("Teenage∗ with cancer")) OR ("Child∗ with cancer")) OR ("Pediatric malign∗ diseas∗")) AND (((((((((((((("Pediatric Palliative Care") OR ("Pediatric Palliative Care Concept")) OR ("Palliative Nursing Care")) OR ("Palliative Nursing")) OR ("Terminal care")) OR ("Palliative Therapy")) OR („Cancer palliative Therapy")) OR ("Palliative Medicine")) OR („Pediatric and Palliative care")) OR („Pediatric nursing")) OR („Pediatric end-of-life care")) OR ("Pediatric Palliative care nursing")) OR ("Hospice care cancer")) OR ((("Palliative Care"[Mesh]) OR "Hospice and Palliative Care Nursing"[Mesh]) OR "Palliative Medicine"[Mesh]))) AND (("Pediatrics"[Mesh]) OR (((((((("Child∗ Oncology") OR ("Pediatric cancer unit")) OR ("Childrens cancer ward")) OR ("Pediatric cancer ward")) OR ("Oncology ward in the clinic")) OR ("Pediatric Oncology unit")) OR ("Pediatric Hematology and Oncology")) OR ("Pediatric oncology ward")))	202
	Filter	• 2014-2025 • Article Language: German and English	141

The arterisk represents a truncation operator used in database searching. Appended to a word root, it retrieves all suffix variants of that term (e.g. nurs yield nurse, nursing, nurses...), thereby maximizing search sensitivity and ensuring comprehensive literature retrieval.

### Study Selection

After removal of duplicates, titles and abstracts were screened independently by 2 reviewers following JBI recommendations.^12^ Full texts were subsequently assessed against eligibility criteria. Disagreements were resolved by discussion or third-party consultation.

### Data Charting and Synthesis

Data extraction followed JBI guidance.^12^ No critical appraisal was conducted because this is a scoping review. A standardized extraction sheet was developed to capture study characteristics (author, year, country, design, setting, population, and aims) and findings related to PPC design, implementation, and evaluation. Extracted data were synthesized narratively and organized according to these categories. Barriers and facilitators were identified inductively and grouped thematically, in line with approaches used in previous scoping reviews.^11^

### Deviations from the Registered Protocol

This scoping review was conducted in accordance with a previously registered protocol^15^ (OSF: https://osf.io/ks5h3/). However, during the course of the work, several conceptual and methodological adjustments were made that deviate from the original plan. Originally, the review aimed to analyze the current state of PPC for children with oncological diseases in Germany. Owing to the very limited number of relevant studies, the scope was expanded to include international studies. In this context, the research questions were also adapted: Instead of solely mapping the existing evidence and challenges, the review focused on the design, implementation, and evaluation of PPC models, as well as barriers and facilitators to their integration into pediatric oncology.

In addition, the search strategy was modified: Instead of the initially planned database LIVIVO, Web of Science was included because it offered broader coverage of international publications. The planned search period (2014-2025) was restricted to 2014-2025, because the search was completed before April 2025. The analysis of the included studies was conducted not—as originally planned—using thematic analysis according to Braun and Clarke, but rather by means of a narrative synthesis structured along predefined thematic categories (design, implementation, evaluation, barriers) to adequately address the heterogeneity of the evidence base. A tabular overview of all deviations from the protocol is provided in Table II.

**Table II:** Deviations between protocol and review (own representation)

Section	As planned in protocol	As conducted in review	Rationale for change
Topic/aim	Focus on Germany: Implementation of palliative care for children with oncological diseases in Pediatric oncology; Aim: assess current state of care, challenges and research gaps	Focus international: Integration of PPC in oncology worldwide; Aim: design, implementation and evaluation of models in inpatient oncology settings	Field turned out to be internationally shaped; very few German studies available and expansion was necessary to answer the research question
Research questions	(1) Which empirical studies exist? (2) What scientific evidence exists on challenges of PPC in Pediatric oncology?	Focused on: design, implementation, evaluation of PPC, and barriers/facilitators	Structured subquestions were necessary for systematic synthesis of heterogeneous literature
Databases	PubMed (Medline), CINAHL, PsycINFO, and LIVIVO	PubMed (Medline), CINAHL, PsycINFO, and Web of Science	LIVIVO yielded very few hits; Web of Science provided broader international coverage
Search period	2014-2025	2014-2023	Review was completed before April 2025; 2024/2025 data not yet available
Context/setting	Inpatient pediatric oncology in Germany	Inpatient pediatric oncology worldwide	Very few studies from Germany; international scope needed to capture sufficient studies
Eligibility criteria	Children/adolescents ≤18 years with cancer, inpatient in Germany, German/English	Children/adolescents ≤18 years with cancer, inpatient worldwide, German/English	Geographic expansion was required to identify a sufficient number of studies
Analysis approach	Thematic analysis according to Braun and Clarke (2022)	Narrative synthesis along predefined categories (design, implementation, evaluation, barriers)	High heterogeneity made formal thematic analysis less appropriate; narrative synthesis was more suitable

## Results

### Study Selection

The search yielded 1277 records. After removal of 731 duplicates, 546 titles and abstracts were screened. Of these, 64 full-text articles were assessed for eligibility, and 34 studies were included in the final synthesis (Figure).

**Figure:**
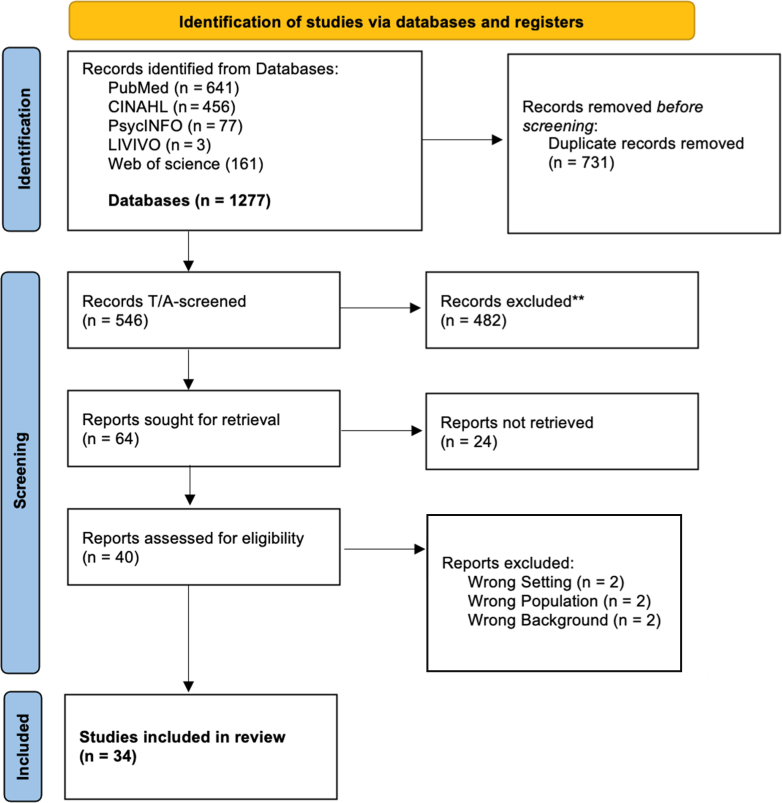
PRISMA flow diagram (Page et al., 2021).

### Study Characteristics

The 34 included studies were published between 2014 and 2025 and originated from 18 countries across Europe, North America, South America, and Asia, as well as from multicountry settings. Most studies used qualitative designs (n = 12), followed by retrospective chart reviews (n = 7), surveys (n = 4), and reviews or guidelines (n = 11). Sample sizes ranged from single-site qualitative interviews to large multicountry surveys. Detailed study characteristics, including study aims, design, and main findings, are provided (Table III).

**Table III:** Characteristics of included studies (own representation)

Author(s), year	Country	Study design	Setting	Population	Aim	Key findings
Valadares et al., 2014	Brazil	Retrospective descriptive	Pediatric oncology ward	29 hematology-oncology patients	Evaluate PPC approaches	Minimal PPC use, poor pain and symptom documentation.
Weaver et al., 2015	USA	Systematic review and consensus	Pediatric/adolescent oncology (global)	—	Recommendations for PPC integration	Supports early PPC, comprehensive family support.
Weaver et al., 2016	USA	Integrative review	Pediatric/adolescent oncology (psychosocial)	—	Inform psychosocial PPC guidelines	Provides standards and evidence map for psychosocial support.
Ferrell et al., 2017	USA	Expert panel guideline update (ASCO)	Oncology (guideline context)	—	Update ASCO guideline on palliative care integration	Supports early PPC integration into standard oncology care.
Kaye et al., 2017	USA	Narrative review	Pediatric oncology (literature)	—	Review models/strategies for PPC integration	Embedded PPC clinics facilitate early integration.
Levine et al., 2017	USA	Cross-sectional survey	Clinic/inpatient oncology	129 dyads (patients aged 10-17 years)	Assess symptom burden and PPC attitudes	High early symptom burden, support for early PPC.
Haines et al., 2018	USA	Conceptual review	Pediatric oncology (literature)	—	Synthesize barriers and strategies	Barriers at the policy, system, organizational, and individual levels.
Ho et al., 2018	Hong Kong	Retrospective 10-year review	Tertiary oncology unit	695 patients; 180 deaths	Assess PPC referrals/services	Low referral rates, late PPC involvement.
Jagt-van Kampen et al., 2018	The Netherlands	Retrospective cohort	Hospital-based PPCT	43 children	Describe PPCT case management	Substantial workload, some differences malignant vs nonmalignant.
Nyirő et al., 2018	Hungary	Qualitative interviews	National oncology centers	22 physicians	Explore barriers to PPC	Barriers: stigma, communication challenges, and limited resources.
Rost et al., 2018	Switzerland	Retrospective registry review	Pediatric oncology registry	193 deceased patients	Assess timing of PPC, decision-making	PPC initiated late, limited child participation.
Spruit and Prince-Paul, 2018	USA	Narrative review	Pediatric oncology (literature)	—	Overview of PPC services/barriers	Underuse and late involvement persist.
Stutz et al., 2018	USA	Retrospective EHR study	Academic hospital	233 deceased children	Analyze PPC consultation patterns	Consults linked to better EOL preparation, variable use.
Szymczak et al., 2018	USA	Qualitative interviews	Large children's hospital	16 providers	Explore perceptions of PPC service	Perceptions/stigma influenced consultation timing.
Cheng et al., 2019	Multicountry	Systematic review and meta-analysis	Pediatric oncology populations	—	Assess timing of PPC initiation	PPC discussions typically near EOL, substantial variation.
Sawin et al., 2019	USA	Qualitative focus groups	Three pediatric hospitals	11 nurse managers	Explore managers' perspectives on communication	Organizational support influences communication.
Brock et al., 2020	USA	Retrospective cohort	Embedded PPO clinic	426 patients	Evaluate PPO clinic model	Earlier referrals, fewer hospital days post implementation.
Ehrlich et al., 2020	Eurasia (11 countries)	Survey	Pediatric oncology services	424 physicians	Analyze obstacles to PPC integration	Late referrals, lack of guidelines and resources across countries.
Rost et al., 2020	Switzerland	Qualitative (focus groups)	Pediatric oncology units	29 providers (nurses, physicians, psycho-oncologists, social worker)	Identify barriers to PPC	Training deficits, unclear responsibilities, insufficient staffing.
Sisk et al., 2020	USA	Qualitative interviews	Three oncology centers	78 parents	Identify communication functions	Families need honest, empathic dialogue.
Bhat et al., 2021	India	Retrospective audit	Pediatric oncology service	Pediatric oncology patients	Analyze referral patterns/triggers	Referrals late, gaps in integration and EOL planning.
Pyke-Grimm et al., 2021	USA	Narrative review	Pediatric oncology (literature)	—	Describe palliative, concurrent, and hospice care	Barriers to access, billing/coverage challenges.
Benini et al., 2022a	International	Consensus and review (GO-PPaCS)	PPC across settings	—	Update international PPC standards	Defines standards for organization, training, quality.
Benini et al., 2022b	International	Narrative review	Pediatric oncology (literature)	—	Review value/application of PPC	Supports early, family-centered PPC, implementation models.
Gillipelli et al., 2022	USA and Guatemala	Qualitative interviews	Two oncology centers	83 providers	Explore PEWS and provider-family communication	Varied communication practices, resource dependent.
Vasli et al., 2022	Iran	Concept analysis	Pediatric oncology (literature)	19 articles	Analyze PPC concept	Clarifies attributes, preconditions, family-centered interdisciplinary care.
Delemere et al., 2023	Ireland	Qualitative interviews	Pediatric oncology context	21 participants (parents, HCPs, volunteers)	Explore needs/challenges	Unmet psychosocial needs, gaps in services, need for coordination.
Ebadinejad et al., 2023	Iran	Qualitative interviews	Pediatric oncology wards	Nurses, parents, children, stakeholders	Explain nurses' strategies for PPC	Adaptive strategies under constraints, need for training/resources.
Pouy et al., 2023	Iran	Qualitative interviews	Oncology department	22 participants	Explore factors to improve nurses' PPC role	Need for training, protocols, collaboration.
Siddiqui et al., 2023	LMICs	Narrative review	Pediatric oncology in LMICs	—	Describe PPC evolution, needs, outcomes	Major gaps in access, training, and policy.
Koyu et al., 2024	Turkey	Qualitative (photovoice)	Oncology wards	16 nurses	Explore communication needs at EOL	Communication challenges, need for structured support.
Mohammadi et al., 2024	Iran	Qualitative descriptive	Pediatric oncology units	21 nurses	Explore ethical challenges in PPC	Ethical tensions, lack of guidance, training needs.
Salamon et al., 2024	Hungary	Mixed-methods (survey and interviews)	Oncology wards	73 HCPs	Assess PPC perceptions and hospice education	Education improved attitudes, identified gaps.
Lacerda et al., 2024	Multiple European countries	Cross-sectional survey	Pediatric oncology centers and PPC services	PPC leads and healthcare professionals from pediatric oncology and PPC services across Europe	To assess the availability, organization, and level of integration of PPC services within pediatric oncology across Europe	Marked heterogeneity in PPC service provision across Europe; wide variation in availability of specialized PPC teams, referral pathways, and institutional integration into pediatric oncology; many centers lacked formalized collaboration and standardized models for early PPC involvement

*ASCO*, American Society of Clinical Oncology; *EOL*, end of life; *HCP*, healthcare provider; *LMIC*, low- and middle-income countries; *PEWS*,Pediatric Early Warning Systems.

The findings are presented according to the predefined review subquestions, which focused on (1) the design of PPC services, (2) the implementation of PPC in pediatric oncology inpatient settings, (3) the evaluation of PPC services, and (4) barriers and facilitators to PPC integration. This structure guided both data extraction and synthesis.

### Design of PPC Services

Several studies described efforts to establish PPC models within pediatric oncology. These included interdisciplinary, hospital-based PPC teams that supported oncology units on consultation^9^^,^^16^, embedded PPC clinics directly integrated into oncology services^9^, and consensus-based international standards outlining key components of PPC.^1^^,^^8^^,^^17^ However, most services lacked standardized referral criteria, care pathways, or institutional policies, and the design and scope of PPC programs varied substantially across countries and individual institutions.

### Implementation of PPC Services

Implementation of PPC was often delayed^14^^,^^18^, ^19^, ^20^ and typically initiated only at the end of life.^13^^,^^21^^,^^22^ In many cases, families were insufficiently involved in decision-making, and communication gaps between professionals and families were described across diverse healthcare settings.^18^, ^19^, ^20^^,^^23^, ^24^, ^25^, ^26^ A recent European cross-sectional survey by Lacerda et al^16^ further demonstrated substantial heterogeneity in the availability, organization, and level of integration of PPC services across pediatric oncology centers, highlighting wide variation between countries and regions.

### Evaluation of PPC Services

Few studies systematically evaluated the outcomes of PPC interventions. Notable exceptions included evaluations of embedded PPC clinics, which were associated with earlier referrals, improved symptom control, and reduced hospital days.^9^^,^^28^^,^^29^ Retrospective chart reviews also suggested that PPC consultations were linked to improved end-of-life preparation and care planning.^30^ However, overall, evidence on evaluation metrics remains scarce, with heterogeneous outcome measures and a lack of longitudinal or comparative data, limiting the ability to draw firm conclusions on effectiveness.

### Barriers and Facilitators to PPC Integration

Across the included studies, 7 major thematic barriers to the integration of PPC in pediatric oncology were identified. The most frequently reported barrier was the lack of standardized service models and institutional guidelines, resulting in highly variable care structures and inconsistent referral criteria.^1^^,^^16^^,^^31^, ^32^, ^33^, ^34^ Closely related were structural barriers, including insufficient staffing, lack of protected PPC positions, limited or absent reimbursement mechanisms, and the lack of formalized referral pathways and institutional policies supporting PPC integration.^27^^,^^33^^,^^35^, ^36^, ^37^ Reported barriers included limited staffing and unstable funding structures^24^^,^^33^, insufficient training and preparedness among oncology professionals^37^^,^^38^, and persistent misconceptions of PPC as synonymous with end-of-life care.^6^

Another consistently mentioned barrier concerned limited formal training in PPC among pediatric oncologists, nurses, and psychosocial professionals, including insufficient preparation in symptom management, communication about prognosis and goals of care, and interdisciplinary collaboration.^39^^,^^40^ Many providers lacked specific PPC knowledge and held persistent misconceptions equating PPC with end-of-life care, which contributed to delayed referrals.^6^^,^^38^ Insufficient communication and limited family participation in decision-making further impeded timely integration and often left families unprepared for the transition to palliative care.^23^, ^24^, ^25^

In addition, several studies described cultural and emotional barriers, including a reluctance to discuss death and dying with families, as well as psychosocial burdens on staff, often exacerbated by inadequate institutional support structures.^34^^,^^41^, ^42^, ^43^ Finally, there was a lack of robust evaluation data on the effectiveness and outcomes of PPC interventions, which hampers the development of evidence-based service models.^9^^,^^30^

Together, these barriers reflect a complex interplay of structural, professional, and cultural factors that hinder the timely and systematic integration of PPC into pediatric oncology care.

## Discussion

### Organizational Models for PPC Integration

The literature does not describe a single universally applicable approach to integrating PPC into pediatric oncology. Instead, 3 recurrent organizational models were identified: embedded PPC services, trigger-based referral systems, and tiered care models. Importantly, the included studies not only described potential benefits, but also highlighted implementation challenges and structural preconditions that determine whether these models can function in practice.

### Embedded PPC Models

Embedded palliative oncology clinics or consultation teams integrated within oncology departments were frequently associated with earlier referrals, improved communication, and better symptom management. However, the literature consistently indicates that this model is highly resource dependent. Successful implementation requires dedicated PPC staff, protected consultation time, and institutional support structures. Several studies reported that limited staffing, lack of reimbursement mechanisms, and unclear role delineation between oncology and palliative care teams hinder sustained operation of embedded services. Furthermore, oncology clinicians sometimes perceived embedded PPC involvement as a transition to end-of-life care, which could delay referrals despite structural availability. Thus, although embedded models may facilitate integration, they appear to be feasible primarily in well-resourced tertiary centers and may be difficult to transfer to smaller institutions.

### Trigger-based Referral Systems

Referral systems based on predefined clinical triggers (eg, relapse, high symptom burden, intensive care unit admission, or poor prognosis) were described as mechanisms to reduce variability in referral decisions. Nevertheless, the literature suggests that trigger systems depend heavily on clinician acceptance and institutional culture. Studies reported that clinicians occasionally override triggers owing to prognostic uncertainty or concern about diminishing parental hope. In addition, the absence of standardized documentation procedures and electronic screening tools limits consistent application. Without staff training and a shared understanding of PPC goals, trigger-based approaches may remain formal policies without practical impact.

### Tiered or Stepped PPC Models

Tiered models distinguish between primary palliative care delivered by oncology professionals and specialist PPC consultation for complex cases. This approach is frequently proposed as particularly relevant in settings with limited specialist resources. However, the main limitation described across studies concerns workforce competencies. Many oncology professionals reported insufficient training in communication, symptom management, and advance care planning. Without systematic education and ongoing support, primary PPC responsibilities may be perceived as an additional burden rather than an integrated component of oncology care. Consequently, tiered models require substantial educational infrastructure and interdisciplinary collaboration to be operational.

### Synthesis

Across all models, the same underlying determinants repeatedly emerged: availability of trained staff, institutional policies, financing structures, and professional attitudes toward palliative care. The literature therefore suggests that PPC integration is less dependent on selecting a specific organizational model than on addressing structural and cultural conditions within healthcare systems. Organizational models provide a framework for integration, but their effectiveness appears contingent upon implementation context rather than the model itself.

### Limitations

This review has several limitations. First, only studies published in English or German were included, which may have excluded relevant evidence in other languages. Second, the review focused exclusively on inpatient oncology settings; outpatient and home-based PPC services were not addressed. Third, the heterogeneity of study designs and outcomes precluded formal comparison or meta-analysis. Finally, although the search strategy was comprehensive, the reliance on 4 databases and the restriction to the period 2014-2025 may have omitted earlier or nonindexed studies.

## Conclusions

PPC is essential for children with cancer and their families, yet its integration within hospital-based oncology services remains inconsistent and often delayed. This scoping review identified substantial gaps in standardized service models, implementation strategies, and evaluation frameworks, alongside 7 recurring barriers that hinder effective integration in inpatient care. To ensure equitable and timely access, health systems should prioritize early, family-centered PPC supported by adequate resources, multidisciplinary training, and structured evaluation processes. Strengthening international collaboration and developing adaptable, context-specific models are crucial to advancing PPC delivery in pediatric oncology. Future research should explore how these strategies can be extended and adapted to outpatient and community-based settings, promoting continuity of care across the disease trajectory.

## Future Research

Future research should explore how strategies can be extended and adapted to outpatient and community-based settings to promote continuity of care throughout the disease course. It should focus on developing and testing standardized PPC models tailored to pediatric oncology, with particular attention to early integration and cultural adaptation. Rigorous evaluation using consistent outcome measures—including quality of life, symptom burden, family satisfaction, and health service utilization—is essential. Comparative studies across different health systems, particularly in low- and middle-income countries, are needed. Research should also explore the perspectives of children themselves and evaluate interventions that strengthen communication and shared decision-making within oncology care.

## CRediT authorship contribution statement

**Stefanie Stober:** Writing – original draft, Visualization, Software, Resources, Project administration, Methodology, Investigation, Formal analysis, Data curation, Conceptualization. **Sebastian Hoffmann:** Writing – review & editing, Visualization, Validation, Resources, Methodology, Formal analysis, Data curation. **Sabine Metzing:** Writing – review & editing, Supervision, Resources, Methodology.

## Declaration of Competing Interest

This review is part of a dissertation project (PaVKo) within the doctoral program in Nursing Science at Witten/Herdecke University. This research did not receive any specific grants from funding agencies in the public, commercial or non-profit sector.
